# Enhanced therapeutic efficacy of doxorubicin against multidrug-resistant breast cancer with reduced cardiotoxicity

**DOI:** 10.1080/10717544.2023.2189118

**Published:** 2023-03-15

**Authors:** Tianyu Zhang, Nuannuan Li, Ru Wang, Yiying Sun, Xiaoyan He, Xiaoyan Lu, Liuxiang Chu, Kaoxiang Sun

**Affiliations:** a Key Laboratory of Molecular Pharmacology and Drug Evaluation (Yantai University), School of Pharmacy, Collaborative Innovation Center of Advanced Drug Delivery System and Biotech Drugs in Universities of Shandong, Ministry of Education, Yantai University, Yantai, China; b Yantai Saipute Analyzing Service Co. Ltd, Yantai, Shandong Province, China

**Keywords:** Cardiotoxicity, multidrug resistance, active targeting, pH-sensitive, immunogenic cell death (ICD)

## Abstract

Doxorubicin (DOX), a commonly used anti-cancer drug, is limited by its cardiotoxicity and multidrug resistance (MDR) of tumor cells. Epigallocatechin gallate (EGCG), a natural antioxidant component, can effectively reduce the cardiotoxicity of DOX. Meanwhile, EGCG can inhibit the expression of P-glycoprotein (P-gp) and reverse the MDR of tumor cells. In this study, DOX is connected with low molecular weight polyethyleneimine (PEI) via hydrazone bond to get the pH-sensitive PEI-DOX, which is then combined with EGCG to prevent the cardiotoxicity of DOX and reverse the MDR of cancer cells. In addition, folic acid (FA) modified polyethylene glycol (PEG) (PEG-FA) is added to get the targeted system PEI-DOX/EGCG/FA. The MDR reversal and targeting ability of PEI-DOX/EGCG/FA is performed by cytotoxicity and *in vivo* anti-tumor activity on multidrug resistant MCF-7 cells (MCF-7/ADR). Additionally, we investigate the anti-drug resistant mechanism by Western Blot. The ability of EGCG to reduce DOX cardiotoxicity is confirmed by cardiotoxicity assay. In conclusion, PEI-DOX/EGCG/FA can inhibit the expression of P-gp and reverse the MDR in tumor cells. It also shows the ability of remove oxygen free radicals effectively to prevent the cardiotoxicity of DOX.

## 1. Introduction

Breast cancer, with the highest morbidity and mortality, is the second reason of cancer-related deaths among women worldwide (Koual et al., 2020). The incidence of breast cancer in Chinese women has been increasing rapidly and getting younger in the past two decades (Fischer et al., 2022). At present, chemotherapy is still one of the common methods for breast cancer therapy in clinical (Palmeira et al., 2012). For example, doxorubicin (DOX), which is commonly used in clinic, has shown good therapeutic effect on breast cancer. In addition to this, it has also been proved to induce immunogenic cell death (ICD) in tumor cells (Li et al., 2021; Ren et al., 2021; Zhang et al., 2022). However, DOX can also cause damage to normal body cells (Varela-López et al., 2019). In particular, due to its affinity for cardiomyocytes, DOX can cause fibrosis and apoptosis of cardiomyocytes via producing oxygen free radicals (Renu et al., 2018). In addition, MDR is also one of the reasons limiting the clinical application of DOX (Matsunaga et al., 2015). The MDR is mainly related to the over-expression of P-glycoprotein (P-gp) in tumor cells, which is a drug transporter encoded by MDR1 (ABCB1) gene and can expel DOX from cells, resulting in reduced anti-tumor effect of DOX. It has also been proved that DOX can induce P-gp expression in tumor cells, further leading to drug resistance of tumor cells (Satonaka et al., 2017).

Epigallocatechin gallate (EGCG), a natural antioxidant component (Gan et al., 2018) which can be used as an antioxidant in various food additives, has also been used in the treatment of anti-tumor, cardiovascular diseases and other diseases (Wang et al., 2018). It can effectively remove oxygen free radicals in the body (Cheng et al., 2016). In addition to this, studies also show that EGCG can significantly inhibit the expression of P-gp (Satonaka et al., 2017). Therefore, the combination of DOX and EGCG can effectively prevent the cardiotoxicity of DOX and reverse the MDR in tumor cells. However, since EGCG is easily oxidized even under common physiological condition (Ziaunys & Smirnovas, 2022), it is critical to design a method that can not only protect the EGCG molecule from oxidation, but also effectively transport EGCG and DOX to the action site.

Tumor microenvironment, with rapid proliferation and differentiation of cancer cells, displays various differences with normal body environment, such as lower pH, higher concentration of reducing substance glutathione (GSH), excessive expression of specific enzymes (Fathi et al., 2018), as well as the enhanced permeability and retention (EPR) effect. Based on the characteristics of tumor microenvironment, many nanoparticles have been reported to passively target tumors by EPR effect (Jin et al., 2019). More effective treatment can be achieved by using stimuli-responsive carriers like the ones with hydrazone bond. The hydrazone bond is stable under neutral conditions but easy to break under acidic or weak acidic conditions (Hu et al., 2018), so that the drugs can be effectively released at the tumor site, resulting an improved therapeutic efficacy and reduced side effect. In addition to the passive targeting delivery and stimuli-responsive release of therapeutic drugs based on the tumor microenvironment, some active targeting delivery systems are also designed based on the special characteristics of tumor cells. Folic acid receptor is a glycoprotein receptor, which has low expression in normal body cells and high expression in breast cancer cells (Choi et al., 2018). It has a high affinity for both folic acid (FA) and folic acid complex (Heo et al., 2019). Therefore, FA modification can effectively improve the active targeting effect of nano-system and deliver drugs to the tumor tissue, improving the drug accumulation at the tumor site while reducing the damage of drugs to normal body cells.

**Figure 1.**
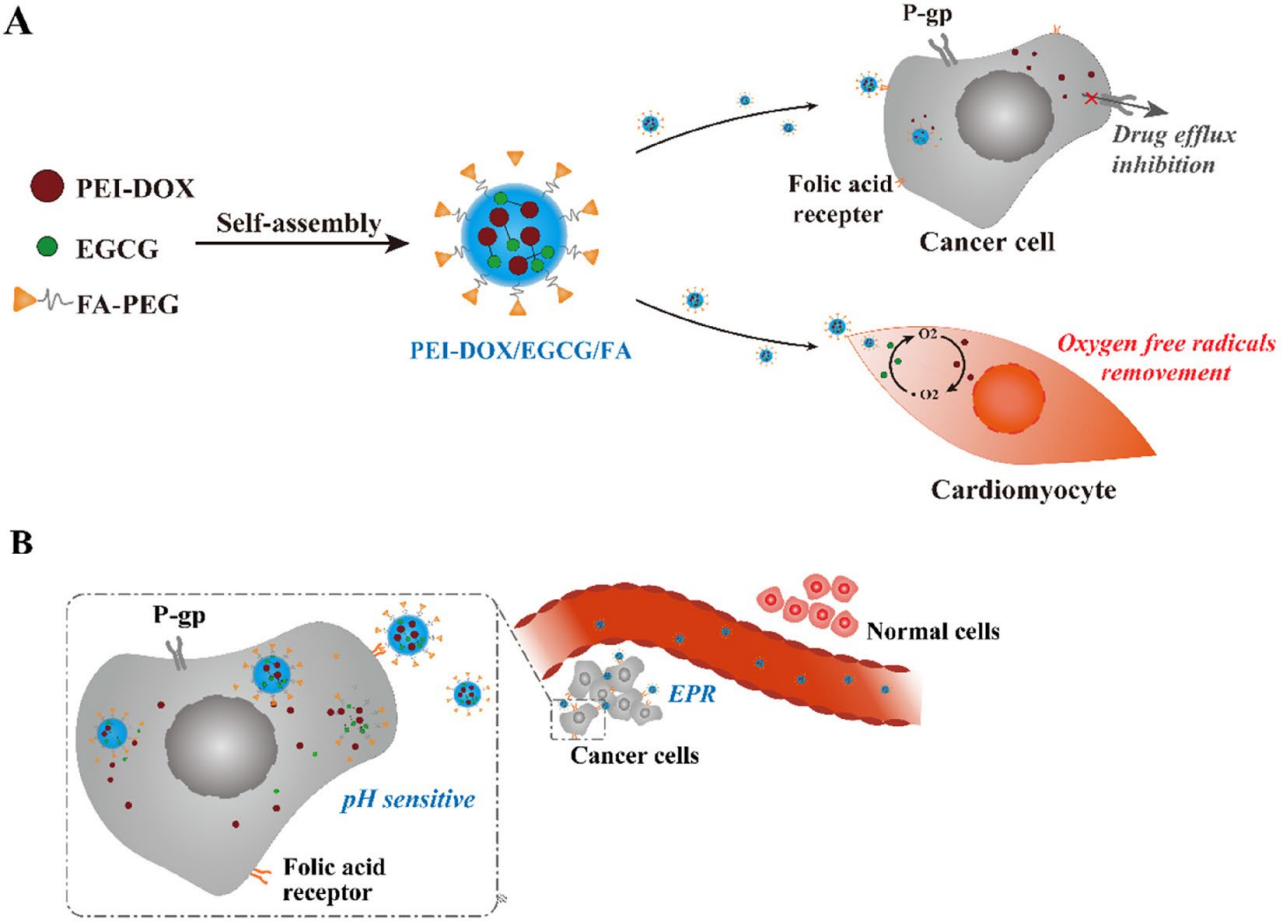
(A) The preparation and mechanism of PEI-DOX/EGCG/FA to overcome multidrug resistance of tumor cell and reduce cardiotoxicity. (B) The targeting mechanism of PEI-DOX/EGCG/FA to tumor cells.

Therefore, to improve the accumulation of DOX and EGCG in tumor sites, and thus reducing the cardiotoxicity of DOX and reversing the MDR of breast cancer cells, we construct an active targeting nano-system. In this system, DOX is firstly connected with low molecular weight polyethyleneimine (PEI) by a hydrazone bond, and then EGCG is added and combined with DOX by π-π conjugation and with PEI via electrostatic interaction, getting the pH-sensitive system PEI-DOX/EGCG. To make the system with active targeting ability, the synthesized PEG-FA is also added. The pH-sensitive and FA-targeting system PEI-DOX/EGCG/FA can deliver EGCG and PEI-DOX to tumor site via active targeting effect, and then the DOX can be released in a pH-sensitive manner. Both the pH-sensitivity and FA-targeting ability improve the DOX accumulation in tumor site, enhancing the antitumor effect and reducing the cardiotoxicity of DOX. In addition to this, the added EGCG can also prevent the cardiotoxicity of DOX while reverse the MDR of cancer cells by inhibiting the P-gp expression level, thus improve the antitumor effect and reducing the cardiotoxicity of DOX in a further step (Figure 1).

## 2. Materials and methods

### 2.1. Materials

FA, PEG_5000_-NH_2_, 1-(3-dimethylaminopropyl)-3-ethylcarbodiimide hydrochloride (EDCI), N-hydroxysuccinimide (NHS) were obtained from Shanghai Aladdin Reagent Co., Ltd. (China). Roswell Park Memorial Institute-1640 (RPMI-1640), fetal bovine serum (FBS), trypsin solution was obtained from Procell Life Science & Technology Co., Ltd. (Wuhan, China). 4% paraformaldehyde solution, 3-(4, 5-dimethylthiazol-2-yl)-2, 5-diphenyltetrazolium bromide (MTT), High mobility group box 1 (HMGB1) Elisa Kit were obtained from Beijing Solarbio Science & Technology Co., Ltd. (Beijing, China). Anti-HMGB1 Rabbit mAb, Anti-Calreticulin Rabbit pAb, Anti-P-glycoprotein Rabbit pAb were purchased from ABclonal Technology Co., Ltd. (Wuhan, China). Annexin V-FITC Apoptosis Detection Kit and Enhanced ATP Assay Kit were provided by Beyotime Biotechnology Co., Ltd. (Shanghai, China). Human Creatine Kinase MB Isoenzyme (CK-MB) ELISA Kit and Malondialdehyde (MDA) Colorimetric Assay Kit (TBA Method) were provided by Elabscience Biotechnology Co., Ltd (Wuhan, China). Lactate dehydrogenase (LDH) assay kit and Total Superoxide Dismutase (T-SOD) assay kit were obtained from Nanjing Jiancheng Bioengineering Institute (Nanjing, China).

### 2.2. Preparation and characterization of nanoparticles

#### 2.2.1. Synthesis and characterization of FA-PEG

PEI-DOX was synthesized by our group according to the previous report (Shi et al., 2014), only FA-PEG was synthesized and character here. In brief, FA was dissolved in DMSO and catalyzed by NHS and EDCI for half an hour at room temperature. And then PEG-NH_2_ (5000) dissolved in ultra-dry DMSO was added to the activated FA reaction solution. After 48 h, the reaction solution was transferred to 1000 Da dialysis bag and dialyzed against deionized water for 2 days. Finally, the dialyzed solution was lyophilized to obtain a yellow powder. ^1^H-NMR and FTIR were used to confirm the structure of FA-PEG.

#### 2.2.2. Preparation and characterization of PEI-DOX/EGCG/FA nanoparticles

PEI-DOX, EGCG and FA-PEG were dissolved in water respectively. EGCG was firstly mixed with PEI-DOX, and FA-PEG aqueous solution was further added to the mixture with stirring. After centrifugation performed with 10000 MWCO ultrafiltration tube at 5000 rpm for 5 min to remove free EGCG and PEI-DOX, PEI-DOX/EGCG/FA nanoparticles were obtained from the upper layer of ultrafiltration tube.

The lower layer of the ultrafiltration tube was tested with Ultraviolet Visible Spectrophotometer (UV, SHIMADZU UV-2450, Japan) to determine the content of DOX at 484 nm, and the content of EGCG was measured by High Performance Liquid Chromatography (HPLC, Agilent 1260GB12C, USA). HPLC detection was performed with 30% v/v methanol, 70% v/v water and 0.1% v/v acid at a flow rate of 1.0 ml/min with diode array detection at 280 nm. The drug loading (DL) and encapsulation efficiency (EE) were calculated as follows. DL (%) = (Total mass of drug dose −  Mass of free drug)/Weight of NPs ×100%. EE (%) = (Total mass of drug dose − Mass of free drug)/Total mass of drug ×100%. The particle size and zeta potential of PEI-DOX/EGCG/FA was measured by Zetasizer Nano ZS (Malvern Instruments, Malvern, United Kingdom). The morphology of PEI-DOX/EGCG/FA was observed by transmission electron microscopy (TEM, JEM-1400 Plus, Jeol, Tokyo, Japan).

#### 2.2.3. In vitro release

The EGCG release from PEI-DOX/EGCG/FA was measured under the condition of pH 5.5 and pH 7.4. PEI-DOX/EGCG/FA solution was firstly transferred into dialysis bag (3500 Da), which was then added into 20 mL PBS (pH 5.5 and pH 7.4) at 37 °C. At fixed time intervals (0.5, 1, 2, 4, 8, 12, 24, 48 h), 1 mL of the release solution was taken out for HPLC test and an equal volume of fresh buffer was added immediately. Detection was performed with 30% v/v methanol, 70% v/v water and 0.1% v/v acid at a flow rate of 1.0 ml/min with diode array detection at 280 nm. The cumulative percentage of EGCG released at different time points was calculated and plotted as a curve.

#### 2.2.4. Stability of EGCG

PEI-DOX/EGCG/FA nanoparticles, diluted by PBS solution (pH 7.4) to make the concentration of EGCG to be 1.0 mg/mL, were placed in 37°C water bath. And then the samples were taken out after incubation of 0, 1, 2, 3 and 4 h. After destroying PEI-DOX/EGCG/FA using DMSO, the EGCG retained in PEI-DOX/EGCG/FA was tested by HPLC. For comparison, the retention of free EGCG was also conducted and tested in the same condition as PEI-DOX/EGCG/FA.

#### 2.2.5. Hemolytic test

The hemolytic test was conducted using 2% red blood cell suspension. Briefly, PEI-DOX/EGCG/FA solutions with DOX concentrations of 0.01, 0.05, 0.1, 0.5 and 1.0 mg/mL were mixed with 2% red blood cell suspension as the experimental group. And the mixture of normal saline with red blood cell suspension was set as negative control group, while that of ultrapure water with red blood cell suspension was set as positive control group. All the samples were incubated at 37°C for 3 h. After centrifugation, the supernatant was collected and the absorbance value of supernatant was measured at 540 nm by UV spectrophotometer, and the hemolysis ratio was calculated.

### 2.3. Cytotoxicity assay

The MCF-7/ADR and MCF-7 cells were seeded at 96-well plate with a density of 5 × 10^3^/well successively and incubated for 12 h. Then, fresh medium containing DOX, PEI-DOX, PEI-DOX/EGCG, PEI-DOX/EGCG/FA with the DOX concentration of 1.0, 5.0, 10.0, 20.0 µg/mL or 0.1, 0.5, 1.0, 2.0 µg/mL were added to MCF-7/ADR or MCF-7 cells. Each sample contains three parallel. After incubated for 48 h, 20 μL MTT solution (5 mg/mL) was added into each well and incubated for another 4 h. And then the medium was removed and 150 μL of DMSO was added until the crystals was dissolved. The absorbance (abs.) of all the samples were measured at 490 nm with microplate reader (Model 550, Bio-Rad, USA). Cell inhibition (%) =1−(Absorbance_Sample_/Absorbance_Control_)×100%.

### 2.4. Mechanism of MDR reversal

The MCF-7/ADR cells were incubated in 6-well plates with a density of 2 × 10^5^/well overnight and then treated with PBS, DOX, PEI-DOX, PEI-DOX/EGCG and PEI-DOX/EGCG/FA at DOX concentration of 1.0 µg/mL, respectively. After incubated for 48 h, the cells were lysed in lysis buffer to collect proteins. Protein quantification by BCA method. The P-gp expression level in MCF-7/ADR cells was detected by Western Blot.

### 2.5. Wound healing assay

The MCF-7/ADR cells were incubated in 6-well plates with a density of 2 × 10^5^/well overnight. After removing the medium, pipetting nozzle was used to draw a straight line on each well, and 2 mL PBS were added to wash off the cell debris. Then, fresh medium containing DOX, PEI-DOX, PEI-DOX/EGCG, PEI-DOX/EGCG/FA with the DOX concentration of 1.0 µg/mL were added. At fixed time intervals (0, 24, 48 h), the wounded area of cells was photographed by microplate reader.

### 2.6. Proliferation inhibition ability on tumor spheroids

150 mg of agarose was dissolved in 10 mL ultra-pure water and sterilized for 30 min at 121°C. Then, 60 μL of agarose solution was added to 96-well plate and cooled to room temperature. Then the MCF-7/ADR cells were seeded at plates. When the 3D tumor spheroids reached the uniform size of 300 μm, PBS, DOX, PEI-DOX, PEI-DOX/EGCG and PEI-DOX/EGCG/FA with DOX concentration of 1.0 µg/mL were added to these MCF-7/ADR spheroids. At fixed time intervals (0, 48, 96 h), the spheroids were photographed by microplate reader.

### 2.7. Cellular uptake

The MCF-7/ADR cells were seeded and incubated for 12 h in 6-well plates. Then, MCF-7/ADR cells were treated with PBS, DOX, PEI-DOX, PEI-DOX/EGCG and PEI-DOX/EGCG/FA at DOX concentration of 1.0 µg/mL. After incubated for another 4 h, collect the cancer cells and resuspended with 500 μL PBS. The fluorescence intensity of DOX taken by MCF-7/ADR cells were detected by flow cytometry.

### 2.8. ICD Validation of DOX

#### 2.8.1. CRT efflux

The MCF-7/ADR cells were incubated in 6-well plates with a density of 2 × 10^5^/well overnight and then treated with PBS, DOX, PEI-DOX, PEI-DOX/EGCG and PEI-DOX/EGCG/FA at DOX concentration of 1.0 µg/mL, respectively. After 24 h, the treated cells were fixed with 4% paraformaldehyde, incubated with anti-CRT rabbit polyclonal antibody (primary antibody), FITC-labeled goat anti-rabbit secondary antibody, and 4′,6-diamidino-2-phenylindole (DAPI), successively. The confocal laser scanning microscope was applied to observe the distribution of CRT.

#### 2.8.2. HMGB1 secretion

The MCF-7/ADR cells were incubated in 6-well plates with a density of 2 × 10^5^/well overnight and then treated with PBS, DOX, PEI-DOX, PEI-DOX/EGCG and PEI-DOX/EGCG/FA at DOX concentration of 1.0 µg/mL, respectively. After 24 h, the treated cells were fixed with paraformaldehyde, added 0.1% TritonX-100 to penetrate the cell membrane. Then incubated with anti-HMGB1 rabbit antibody and FITC-labeled goat anti-rabbit antibody, and stained with DAPI, successively. The confocal laser scanning microscope was applied to observe the fluorescence intensity of HMGB1.

#### 2.8.3. ATP release

The MCF-7/ADR cells were incubated in 6-well plates with a density of 2 × 10^5^/well overnight and then treated with PBS, DOX, PEI-DOX, PEI-DOX/EGCG and PEI-DOX/EGCG/FA at DOX concentration of 1.0 µg/mL, respectively. After 24 h, the culture medium was collected and operated according to the instructions of ATP detection kit.

### 2.9. *In vitro* cardiotoxicity assay

#### 2.9.1. Cardiac cytotoxicity

The H9c2 cells were seeded at 96-well plate with a density of 5 × 10^3^/well and incubated for 12 h. Then, fresh medium with DOX, PEI-DOX, PEI-DOX/EGCG and PEI-DOX/EGCG/FA were added to H9c2 cells. The final concentration of DOX was 0.1, 1.0, 5.0, 10.0, 20.0 µg/mL. Each sample contains three parallel. After incubated for another 48 h, 20 μL MTT solution (5 mg/mL) was added and incubated for another 4 h. And then the medium was removed and 150 mL of DMSO was added to dissolve the crystals. The absorbance (abs.) of all the samples were measured at 490 nm with microplate reader. Cell viability (%) = (Absorbance_Sample_/Absorbance_Control_)×100%

#### 2.9.2. Cardiac damage and oxidative indicators

The H9c2 cells were incubated for overnight in 6-well plates. Then, they were treated with PBS, DOX, PEI-DOX, PEI-DOX/EGCG and PEI-DOX/EGCG/FA at DOX concentration of 1.0 µg/mL. After 48 h, the cells were collected and broken by ultrasonic cell crushing instrument at 200 W power. After centrifuged at 12000 g for 4 min, the supernatant was collected and the content of MDA and total SOD activity were detected according to the instructions of assay kits. Protein quantification by BCA method.

To test the release of LDH and CK-MB into the cell culture medium, the H9c2 cells were incubated for overnight in 6-well plates. Then, they were treated with PBS, DOX, PEI-DOX, PEI-DOX/EGCG and PEI-DOX/EGCG/FA at DOX concentration of 1.0 µg/mL. After 48 h, the culture medium was collected and operated according to the instructions of assay kits for LDH and CK-MB.

### 2.10. *In vivo* anti-tumor activity

About 1.0 × 10^6^ MCF-7/ADR cells were slowly injected into the subcutaneous region of male nude mice. When the tumor volume of mice grew to about 100 mm^3^, dividing them into 5 groups (5 mice per group) randomly: saline group, DOX group, PEI-DOX group, PEI-DOX/EGCG group and PEI-DOX/EGCG/FA group, and the drug was administered with DOX dose of 5 mg/kg every 3 days. Tumor volume were monitored every two days. Two days after the last injection, the nude mice were sacrificed and the tumors were photographed and weighed. Tumor volume (mm^3^) =L_a_*L_b_^2^/2. Tumor inhibition ratio (%) =(W_c_−W_t_)/W_c_. L_a_ and L_b_ are the long and short diameter of tumors; W_t_ and W_c_ are the final tumor weights of nude mice in the treatment and saline groups, respectively.

### 2.11. Tissue distribution

After the last administration, mice were executed. Then, heart and tumor tissues were removed, quantified and tissue homogenized to make a 10% tissue homogenate. DOX fluorescence intensity was measured at 490 nm excitation light and 550 nm emission light using by microplate reader.

### 2.12. Histopathology analysis

Tumors, heart, liver, lung and kidney of mice were selected for histological observation. The organs were dissected, washed in PBS and fixed in 4% paraformaldehyde solution. After paraffin embedding and sectioning, the tissue sections were stained with hematoxylin and eosin (H&E) for histological analyses. Then, cell apoptotic analyses were detected by terminal deoxynucleotidyl transferase dUTP nick end labeling (TUNEL) apoptosis detection kit.

### 2.13. Immunological analysis

The blood of mice was taken and 5000 rpm centrifuged at 4°C for 10 min, then the supernatant was retained. The expression of IFN-γ, TNF-α, IL-10 in serum was measured by ELISA assay kits.

Tumor tissues were collected and broken by ultrasonication, then 14000 rpm centrifuged at 4°C for 10 min. The supernatant was detected by using IFN-γ, TNF-α, IL-10 ELISA assay kits. Protein quantification by BCA method.

### 2.14. *In vivo* expression of P-gp

Tumor tissues were collected and broken by ultrasonication in RIPA Lysis Buffer to collect proteins. Protein quantification by BCA method. The expression of P-gp in tumor was analyzed by Western Blot.

### 2.15. *In vivo* cardiotoxicity assay

#### 2.15.1. Cardiac damage and oxidative indicators

A 10% tissue homogenate was prepared from the heart tissues, and the total protein content was determined by BCA assay, the level of MDA and SOD were detected by the instructions of assay kits. The level of the LDH and CK-MB in serum was detected according to assay kits.

#### 2.15.2. Myocardial tissue fibrosis condition

Heart tissue were collected, then washed in PBS and fixed in 4% paraformaldehyde for 24 h. Masson staining was performed according to the Masson trichrome staining kit.

### 2.16. Statistical analysis

Dates were expressed as mean ± standard deviations (SD). The difference in values between the two groups was tested using the two-tailed Student’s t-test by GraphPad Prism. And a value of *p* < 0.05(**) and *p* < 0.01(***) was considered statistically significant.

## 3. Results and discussion

### 3.1. Preparation and characterization of nanoparticles

#### 3.1.1. Synthesis and characterization of FA-PEG

**Figure 2.**
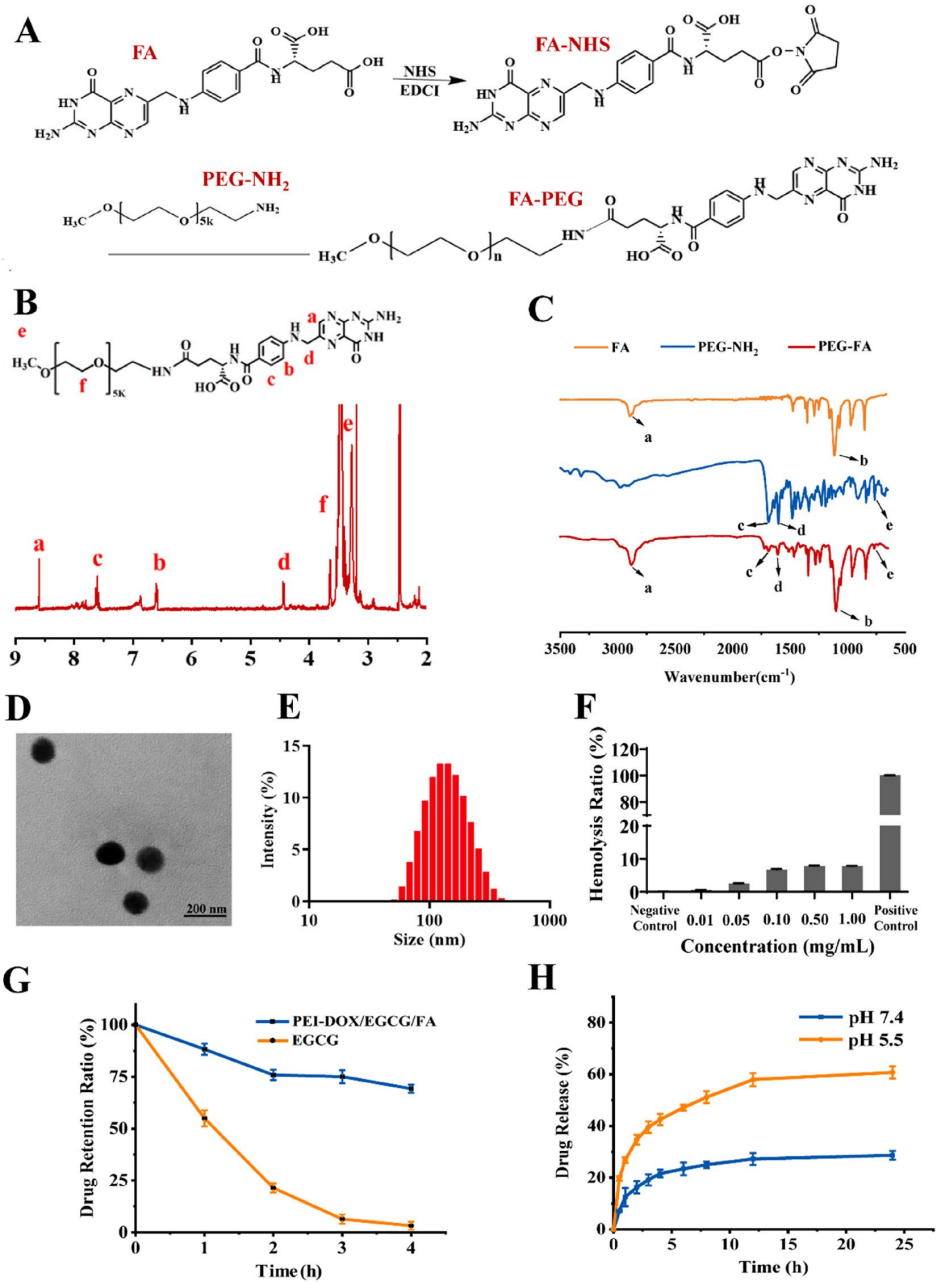
(A) Synthetic route of FA-PEG. (B) ^1^H-NMR spectrum of FA-PEG. (C) FTIR spectra of FA, PEG-NH_2_ and FA-PEG. (D) TEM images of PEI-DOX/EGCG/FA. (E) Particle size of PEI-DOX/EGCG/FA. (F) Hemolysis ratio of free DOX, PEI-DOX, PEI-DOX/EGCG and PEI-DOX/EGCG/FA. (G) EGCG retention ratio in free EGCG and PEI-DOX/EGCG/FA at pH 7.4.(H)In vitro EGCG release at various pH.

FA-PEG was synthesized according to the route in Figure 2(A). FA was firstly activated into FA-NHS using EDCI as a catalyst, and the anhydride of FA-NHS was then reacted with the amine group of PEG-NH_2_ to form the final product FA-PEG. The structures of FA-PEG were determined by ^1^H-NMR and FTIR spectroscopy. Figure 2(B) showed the ^1^H-NMR spectrum of FA-PEG, and the peaks at δ 3.6 ppm and δ 3.2 ppm was ascribed to the groups CH_3_-O-(-CH_2_-CH_2_-O)_5k_ of PEG-NH_2_ while that at δ 7.6 ppm and δ 6.6 ppm were ascribed to the group (-C_6_H_5_-) of FA. The appearance of the characteristic peaks of PEG-NH_2_ and FA in the ^1^H-NMR spectrum of FA-PEG indicated the synthesis of FA-PEG. In the FTIR spectra of FA, PEG-NH_2_ and PEG-FA (Figure 2(C)), PEG-FA showed infrared characteristic peaks of FA (a, b) and PEG-NH_2_ (c, d, e), indicating the FA and PEG were conjugated successfully.

#### 3.1.2. Characterization of PEI-DOX/EGCG/FA nanoparticles

As shown in Figure 2(D), the morphology of PEI-DOX/EGCG/FA was observed by TEM. It could be seen that the PEI-DOX/EGCG/FA nanoparticles were roughly spherical and there was no agglomeration. The zeta potential of PEI-DOX/EGCG/FA was tested to be (−3.43 ± 0.98) mV, and the particle size of PEI-DOX/EGCG/FA was (124.6 ± 1.3) nm with a narrow particle size distribution (PDI < 0.2) (Figure 2(E)). It has been reported that nanoparticles are easily cleared by the kidney when their particle size was less than 5 nm (Xi et al., 2022), and the nanoparticles with a diameter smaller than 200 nm could facilitate the tumor accumulation due to the enhanced permeability and retention (EPR) effect (Peer et al. 2007). Thus, the PEI-DOX/EGCG/FA with suitable size could target tumors without being rapidly cleared by the kidney. The drug loading efficiency of DOX and EGCG in PEI-DOX/EGCG/FA was 5.44% and 62.97%, and the encapsulation efficiency of DOX and EGCG in PEI-DOX/EGCG/FA was 95.89% and 66.63%, respectively.

#### 3.1.3. *In vitro* release

The release experiments were performed in pH 7.4 and pH 5.5 PBS to simulate the normal physiological environment and acidic tumor environment, respectively (Liu et al., 2022; Migliorini et al., 2022). In our study, PEI and DOX were connected by a hydrazone bond, which was easily broken under acidic conditions, resulting in pH sensitivity of PEI-DOX/EGCG/FA. Therefore, EGCG could rapidly release from PEI-DOX/EGCG/FA at pH 5.5. As can be seen from the Figure 2(H), the release of EGCG reaches (60.73 ± 2.41) % at 24 h under the condition of pH 5.5, which was twice as much as that of pH 7.4 ((28.67 ± 1.70) %). These results implicated that the pH-sensitive bond of PEI-DOX/EGCG/FA was broken at pH 5.5, which resulted a high release of EGCG in an acidic tumor environment, reducing the damage of PEI-DOX/EGCG/FA to normal tissues.

#### 3.1.4. Stability of EGCG

Easily occurred auto-oxidation of EGCG under common physiological condition implicated the instability of EGCG (Unno et al., 2021; Ungarala et al., 2022). In the PBS of pH 7.4 at 37°C, the retention ratio of EGCG in PEI-DOX/EGCG/FA could reach (75.86 ± 2.50) % at 2 h (Figure 2(G)), which was three times higher than that of free EGCG (21.45 ± 2.15) %. At 4 h, the retention ratio of free EGCG was only (3.25 ± 1.91) %, while the retention ratio of EGCG in PEI-DOX/EGCG/FA was (69.28 ± 1.96) %. These results proved that PEI-DOX/EGCG/FA nanoparticles could effectively reduce the auto-oxidation and increase the stability of EGCG.

#### 3.1.5. Hemolytic test

Hemolysis ratio was one of the safety indexes of intravenous injection (Lin et al., 2023). As shown in Figure 2(F), the hemolysis ratio of PEI-DOX/EGCG/FA at the highest concentration of 1.0 mg/mL was (7.81 ± 0.05) %, which was lower than 10%, resulting a good biosafety and demand of intravenous administration.

### 3.2. Cytotoxicity assay

**Figure 3.**
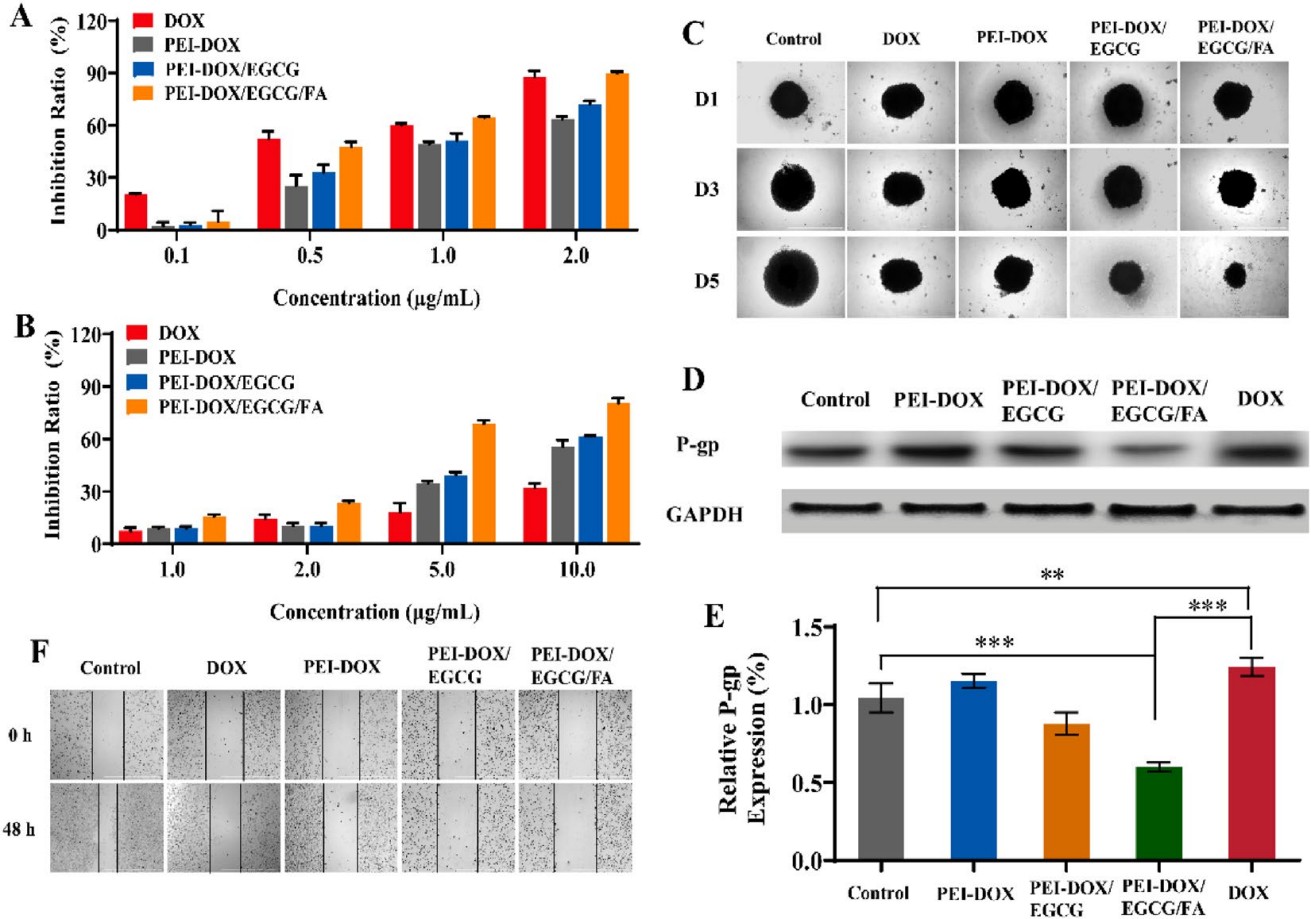
(A) Cytotoxicity of free DOX, PEI-DOX, PEI-DOX/EGCG, PEI-DOX/EGCG/FA in MCF-7 cells. (B) Cytotoxicity of free DOX, PEI-DOX, PEI-DOX/EGCG, PEI-DOX/EGCG/FA in MCF-7/ADR cells. (C) Proliferation inhibition of 3D tumor spheroid treated with free DOX, PEI-DOX, PEI-DOX/EGCG and PEI-DOX/EGCG/FA at days 1, 3, and 5. All images share the same scale bar of 1000 μm. (D) Western Blot results of P-gp expression from MCF-7/ADR cells. (E) The protein expression level of P-gp. (F) MCF-7/ADR cell migration at 0 h and 48 h after treated with free DOX, PEI-DOX, PEI-DOX/EGCG and PEI-DOX/EGCG/FA. All images share the same scale bar of 1000 μm. **p < 0.05, ***p < 0.01.

The cytotoxicity of PEI-DOX/EGCG/FA was conducted on sensitive MCF-7 cells and chemo-resistant MCF-7/ADR cells. It could be seen from Figure 3(A) that all the formulations displayed high anti-cancer effect against MCF-7 cells within the DOX concentration ranging from 0.1 μg/mL to 2.0 μg/mL. In addition, the calculated half-maximal inhibitory concentration (IC_50_) of PEI-DOX/EGCG/FA was (0.53 ± 0.02) μg/mL, which was lower than that of PEI-DOX/EGCG ((0.88 ± 0.03) μg/mL), indicating that FA can improve the active targeted ability of the nanoparticles and enhance the cellular uptake of cancer cells (Patra et al., 2022).

For chemo-resistant MCF-7/ADR cells (Figure 3(B)), the high expression of P-gp resulted in a reduced cytotoxicity of DOX and nanoparticles, especially for free DOX whose inhibition ratio against MCF-7/ADR cells was only (32.03 ± 2.06) % even at the concentration of 10.0 μg/mL. The cytotoxicity of PEI-DOX was slightly higher than that of DOX, which might be due to the structural modifications, resulting in diminished cellular efflux of PEI-DOX. Furthermore, the toxicity of PEI-DOX/EGCG was further increased, probably due to the anti-tumor effect of EGCG, while it was also reported that EGCG could inhibit P-gp expression (Satonaka et al., 2017). In addition, the toxicity of PEI-DOX/EGCG/FA was higher than that of PEI-DOX/EGCG due to the targeting effect of FA. The IC_50_ of PEI-DOX, PEI-DOX/EGCG, PEI-DOX/EGCG/FA were (8.66 ± 0.39) μg/mL, (7.76 ± 0.03) μg/mL, (4.89 ± 0.07) μg/mL respectively.

### 3.3. Mechanism of MDR reversal

To investigate the mechanism of PEI-DOX/EGCG/FA in proliferation inhibition to MCF-7/ADR cells, the P-gp expression level was analyzed by Western Blot. As shown in Figure 3(D)–(E), treatment of DOX in MCF-7/ADR cells resulted in obviously increase in P-gp (*p* < 0.01), while PEI-DOX induced a slight improvement of P-gp expression compared to control. Meanwhile, the expression of P-gp protein in MCF-7/ADR cells treated with PEI-DOX/EGCG and PEI-DOX/EGCG/FA was significantly reduced compared with DOX (*p* < 0.01), indicating that EGCG could inhibit the express of P-gp and reverse the MDR of MCF-7/ADR cells. Besides, since EGCG is easily oxidized and PEI-DOX/EGCG/FA enhances its stability, PEI-DOX/EGCG/FA possesses a better effect of inhibiting P-gp expression than PEI-DOX/EGCG. The EGCG in P-gp suppression together with FA in active targeting facilitated the high anti-tumor efficacy of PEI-DOX/EGCG/FA.

### 3.4. Wound healing assay

In order to determine the efficacy of PEI-DOX/EGCG/FA against cancer cell migration *in vitro* (Zhang et al., 2021), the wound healing assays were introduced. As shown in Figure 3(F), DOX treatment at 1.0 μg/mL had no significant effect in inhibiting MCF-7/ADR cell migration. The inhibition of cell migration by PEI-DOX in combination with EGCG was significantly enhanced compared with that of PEI-DOX, which might due to the P-gp inhibition ability of EGCG. Additionally, the FA-modified PEI-DOX/EGCG/FA with active targeting ability displayed the highest ability in the inhibition of cell migration.

### 3.5. Proliferation inhibition ability on tumor spheroids

To further evaluate the antitumor efficacy of PEI-DOX/EGCG/FA nanoparticles, we examined the effects of DOX, PEI-DOX, PEI-DOX/EGCG, PEI-DOX/EGCG/FA on the proliferation of MCF-7/ADR tumor spheroid. As shown in Figure 3(C), the untreated MCF-7/ADR spheroid showed a rapid growth trend, and DOX and PEI-DOX displayed similar ability in inhibiting the growth of tumor spheroid. Consistent with the results in wound healing assays and cytotoxicity assay, the addition of EGCG made PEI-DOX/EGCG showed higher efficacy in growth inhibition of MCF-7/ADR tumor spheroid, and the PEI-DOX/EGCG/FA demonstrated the highest antitumor efficacy with the assistance of EGCG for P-gp inhibition and FA for active targeting.

### 3.6. Cellular uptake

**Figure 4.**
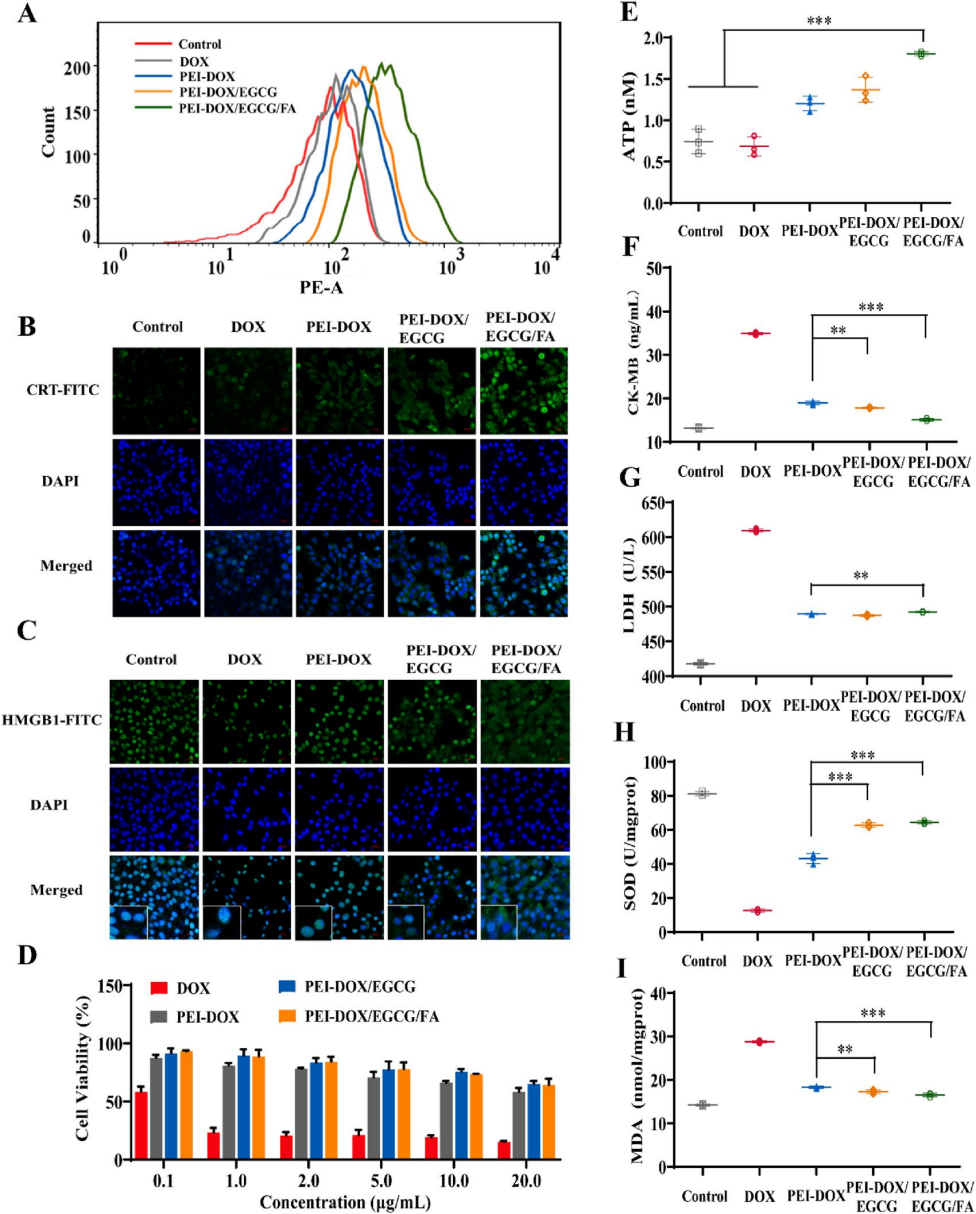
(A) Cellular uptake capacity of free DOX, PEI-DOX, PEI-DOX/EGCG and PEI-DOX/EGCG/FA. (B) The confocal microscopy images of MCF-7/ADR cells co-stained with anti-CRT-FITC (green) and DAPI (blue) of each group. All images share the same scale bar of 20 μm. (C) The confocal microscopy images of MCF-A/ADR cells co-stained with anti-HMGB1-FITC (green) and DAPI (blue) of each group. All images share the same scale bar of 20 μm. (D) Cytotoxicity of free DOX, PEI-DOX, PEI-DOX/EGCG, PEI-DOX/EGCG/FA in H9c2 cells for 48 h. (E) Expression of ATP in MCF-7/ADR cells. (F) Expression of CK-MB after free DOX, PEI-DOX, PEI-DOX/EGCG and PEI-DOX/EGCG/FA treated with MCF-7/ADR cells for 48 h. (G) Expression of LDH after free DOX, PEI-DOX, PEI-DOX/EGCG and PEI-DOX/EGCG/FA treated with MCF-7/ADR cells for 48 h. (H) Expression of SOD after free DOX, PEI-DOX, PEI-DOX/EGCG and PEI-DOX/EGCG/FA treated with MCF-7/ADR cells for 48 h. (I) Expression of MDA after free DOX, PEI-DOX, PEI-DOX/EGCG and PEI-DOX/EGCG/FA treated with MCF-7/ADR cells for 48 h. **p < 0.05, ***p < 0.01.

To compare endocytosis of PEI-DOX, PEI-DOX/EGCG, PEI-DOX/EGCG/FA and free DOX on MCF-7/ADR cells, flow cytometry analysis was performed. It can be seen from the Figure 4(A) that at the DOX concentration of 1.0 μg/mL, PEI-DOX/EGCG/FA could be effectively uptake by MCF-7/ADR cells, indicating that the active targeting ability could be improved after being modified by FA. In addition, the cellular uptake of the PEI-DOX/EGCG was higher than that of PEI-DOX, indicating that EGCG could reverse the drug resistance of tumor cells (Aggarwal et al., 2022) and inhibit the efflux of DOX.

### 3.7. ICD Validation of DOX

#### 3.7.1. CRT efflux

ICD tumor cells can secrete adenosine triphosphate (ATP), translocate calreticulin (CRT) from the endoplasmic reticulum to the cell surface, and release high mobility group protein-1 (HMGB1) from the nucleus. Therefore, DOX-induced ICD effects can be determined by measuring the expression of CRT, HMGB1 and ATP. Immunofluorescence assay was applied to detect the expression level of CRT in MCF-7/ADR cells. It can be seen from Figure 4(B) that the fluorescence intensity in MCF-7/ADR cells treated with DOX and PEI-DOX was much lower than that of PEI-DOX/EGCG and PEI-DOX/EGCG/FA. These results might due to the addition of EGCG and the modification of FA which could prevent the efflux and improve the uptake of DOX, respectively.

#### 3.7.2. HMGB1 secretion

The cancer cells undergoing ICD could expel HMGB1 from nucleus outside the cells (Gao et al., 2022; Turhon et al., 2022). The expression level of HMGB1 on MCF-7/ADR cells was conducted by immunofluorescence assay. As seen in Figure 4(C), HMGB1 in the control group was mainly expressed in the nucleus, but the ones in the treatment groups were expelled because of the ICD effect. Particularly, the HMGB1 expressed in the nucleus of MCF-7/ADR cells treated with PEI-DOX/EGCG/FA was much lower than that of DOX, PEI-DOX and PEI-DOX/EGCG, because of the highest cellular uptake of DOX which could induce ICD (Zhan et al., 2023).

#### 3.7.3. ATP release

The expression of ATP in the supernatant of MCF-7/ADR cells was detected by chemiluminescence (Figure 4(E)). Compared with control (0.74 ± 0.12) nM, the expression of ATP in the supernatant of MCF-7/ADR cells treated with DOX (0.68 ± 0.09) nM did not increase in general, while that treated with PEI-DOX/EGCG/FA increased significantly with (1.80 ± 0.02) nM (*p* < 0.01), demonstrating that PEI-DOX/EGCG/FA could induce an effective ICD effect in MCF-7/ADR cell because of the sufficient cellular uptake of DOX.

### 3.8. Cardiotoxicity assay

#### 3.8.1. Cardiac cytotoxicity

To assess the safety of PEI-DOX/EGCG/FA on myocardial cells, cytotoxicity assay was conducted on the H9c2 cell. As shown in Figure 4(D) that the cell viability of H9c2 treated with DOX was reduced, indicating the severe cytotoxicity of DOX. In addition, PEI-DOX/EGCG and PEI-DOX/EGCG/FA do not show significant myocardial cytotoxicity, which were lower than that of PEI-DOX. This may due to the fact that EGCG can remove the oxygen free radicals which could cause the damage to myocardial cells, reducing the cardiac toxicity of DOX (Shubhra et al., 2021).

#### 3.8.2. Cardiac damage and oxidative indicators

Cardiac enzymes are a general term for a variety of enzymes located in the myocardium, including creatine kinase isoenzymes (CK-MB) and lactate dehydrogenase (LDH). Many diseases that can cause tissue damage can lead to increased LDH activity. CK-MB is mainly stepwise in cardiac myocytes and is a marker of myocardial injury (Jiao et al., 2022; Qi et al., 2023). To detect the safety of PEI-DOX/EGCG/FA on myocardial cells, Therefore, the LDH and CK-MB were tested. It can be seen from Figure 4(F) that compared with the control (4.42 ± 0.31) ng/mgprot, the level of CK-MB in H9c2 cells treated with DOX had increased to (16.22 ± 0.09) ng/mgprot while that of PEI-DOX/EGCG and PEI-DOX/EGCG/FA was only (7.43 ± 0.09) and (5.41 ± 0.36) ng/mgprot, respectively. The detection of LDH shown in Figure 4(G) also displayed the similar result as CK-MB that PEI-DOX/EGCG and PEI-DOX/EGCG/FA exhibited much lower damage to myocardial cells than DOX and even PEI-DOX.

SOD was the main enzyme of the body’s oxygen free radical system, the level of SOD could reflect the body’s ability to remove free radicals. MDA was one of the lipid peroxide reactions in the body, which could indirectly reflect the degree of damage to the body. SOD and MDA often used to detect the degree of antioxidant capacity/oxidation damage of body (Qi et al., 2023). As Figure 4(H)–(I) showed that the level of SOD and MDA in H9c2 cells changed significantly after DOX incubating for 48 h compared with control. By contrast, the level of SOD and MDA in H9c2 cells treated with PEI-DOX/EGCG and PEI-DOX/EGCG/FA did not change visibly. In conclusion, PEI-DOX/EGCG/FA could effectively inhibit the elevated of MDA caused by DOX. At the same time, it also kept SOD vitality at a high level, which could reduce the oxidative damage of H9c2 cells.

### 3.9. *In vivo* anti-tumor activity

**Figure 5.**
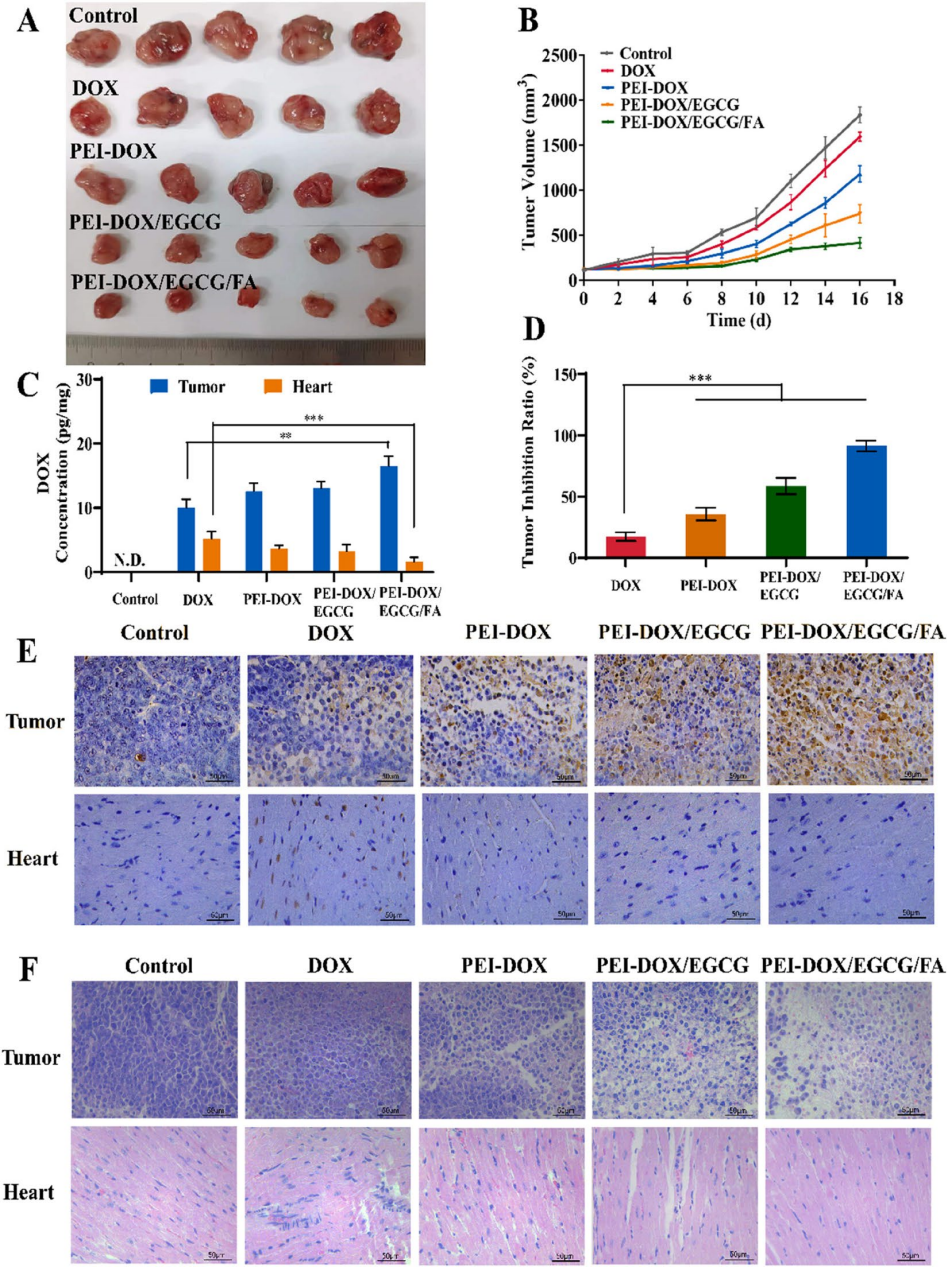
(A) Images of MCF-7/ADR tumors of the mice after treatment. (B) Tumor volume of the mice after treatment. (C) DOX concentration in tumor and heart tissues of mice after treatment. (D) Tumor growth inhibition ratio of DOX, PEI-DOX, PEI-DOX/EGCG and PEI-DOX/EGCG/FA. (E) TUNEL staining of tumor-bearing mice. All images share the same scale bar of 50 μm. (F) H&E staining of tumor-bearing mice. All images share the same scale bar of 50 μm. **p < 0.05, ***p < 0.01.

To assess the anti-tumor efficacy of the PEI-DOX, PEI-DOX/EGCG, PEI-DOX/EGCG/FA *in vivo*, MCF-7/ADR heterotopic tumor-bearing nude mice were used. Figure 5(B) showed that the tumor volume of control group increases by 10 folds from day 0 to day 16. Meanwhile, compared to the control group groups treated with PEI-DOX, PEI-DOX/EGCG and PEI-DOX/EGCG/FA showed much smaller tumor volume, while free DOX could only slightly reduce tumor volume. Furthermore, the tumor inhibition ratio of PEI-DOX/EGCG/FA is 10 folds as much as free DOX (Figure 5(A) and (D)). It indicated that the EGCG in P-gp suppression together with FA in active targeting facilitated the high anti-tumor efficacy of PEI-DOX/EGCG/FA.

### 3.10. Tissue distribution

We measured DOX content in tumor and heart tissues. Figure 5(C) showed that the PEI-DOX group possessed higher DOX concentrations in tumor tissues and lower concentrations in heart tissues than the free DOX group, demonstrating that the modification of pH-sensitive hydrazone bonds can increase DOX concentrations in tumor sites while reducing DOX accumulation in the heart. Furthermore, the PEI-DOX/EGCG/FA group possessed higher DOX concentrations in tumor tissue, which was attributed to the active targeting ability of FA. It proved that PEI-DOX/EGCG/FA can effectively increase the DOX concentration at the tumor site and thus improve the therapeutic efficacy, while reducing DOX accumulation at the heart site and thus reducing cardiotoxicity, compared with free DOX.

### 3.11. Histopathology analysis

The therapeutic effect of the DOX, PEI-DOX, PEI-DOX/EGCG and PEI-DOX/EGCG/FA was evaluated by histological analysis. As Figure 5(E) shown that the PEI-DOX group showed a high extent of apoptosis than DOX in tumor tissues, which proved that the structural changes from DOX to PEI-DOX that led to a lower drug efflux of drug-resistant tumor cells, resulting in an increased extent of apoptosis. Furthermore, the PEI-DOX/EGCG/FA group showed the highest extent of apoptosis in tumor tissues, indicating that PEI-DOX/EGCG/FA can enhance the efficacy of DOX by inhibiting the expression of P-gp with EGCG and proved the targeting ability by FA. At the same time, in heart tissue, PEI-DOX had fewer apoptotic cells due to the PH-sensitive hydrazone compared with DOX, and further reduction of apoptosis in PEI-DOX/EGCG group demonstrated that EGCG removing oxygen free radicals alleviated DOX-induced cardiotoxicity.

To further evaluate the anti-tumor effect and toxicity, H&E staining was performed. It can be seen from the Figure 5(F) that PEI-DOX and PEI-DOX/EGCG caused more tumor cell necrosis than the free DOX group. Furthermore, PEI-DOX/EGCG/FA is more effective in causing tumor cell necrosis by inhibiting the expression of P-gp and proved the targeting ability. Besides, compared with the control group, DOX could cause myocardial tissue damage, while PEI-DOX/EGCG and PEI-DOX/EGCG/FA had no obvious damage compared with the control group. Therefore, PEI-DOX/EGCG/FA can significantly reduce the DOX-induced cardiotoxicity.

### 3.12. Immunological analysis

IL-10 is a suppressive interleukin that mediates anti-tumor immunosuppression *in vivo*. TNF-α is a tumor necrosis factor that has the effect of killing tumor cells and causing tumor cell necrosis. IFN-γ is a signature cytokine of type I helper T cells that has regulatory immune and anti-tumor effects (Kan et al., 2022). Therefore, we used ELISA assay kits to detect changes in cytokines in serum and tumor tissues.

**Figure 6.**
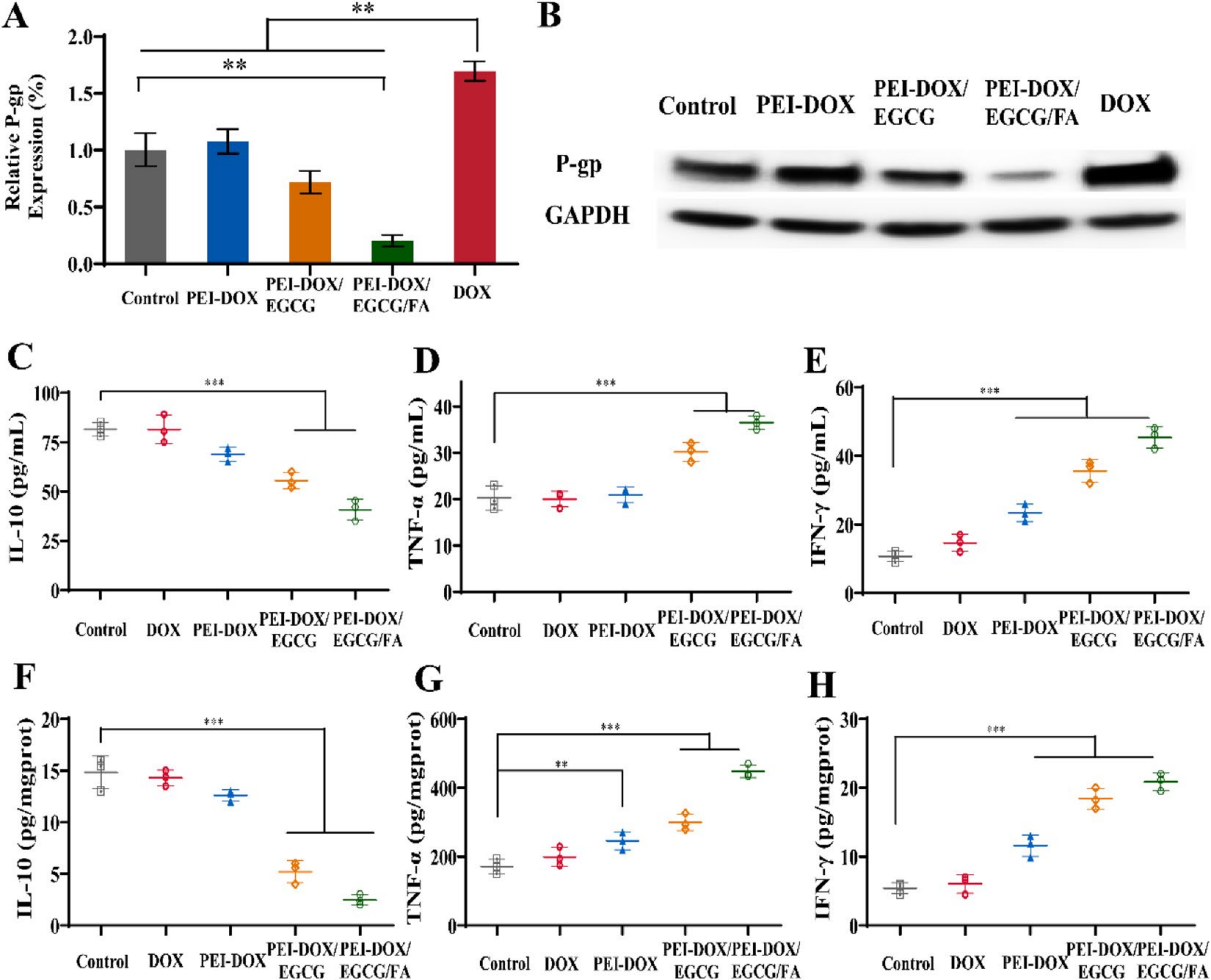
(A) The protein expression of P-gp in tumor. (B) Western Blot results of P-gp expression from tumor tissue. (C) The level of IL-10 in serum. (D) The level of TNF-α in serum. (E) The level of IFN-γ in serum. (F) The level of IL-10 in tumor. (G) The level of TNF-α in tumor. (H) The level of IFN-γ in tumor. **p < 0.05, ***p < 0.01.

Figure 6(C)–(E) shows that the levels of immunosuppressive IL-10 decreased to (68.83 ± 3.55) pg/mL in PEI-DOX group, while the levels of TNF-α and IFN-γ increased to (20.94 ± 1.68) and (23.37 ± 2.51) pg/mL. It probably due to the changed structural of PEI-DOX that leading to a lower drug efflux of drug-resistant tumor cells, resulting in an increased anti-tumor immunity of body. After PEI-DOX/EGCG treated, the level of IL-10, TNF-α and IFN-γ has changed to (55.52 ± 4.09), (30.15 ± 2.02) and (35.65 ± 3.20) pg/mL, respectively. The further enhancement of anti-tumor immunity in PEI-DOX/EGCG group was not only due to the anti-tumor effect of EGCG, but also demonstrated that EGCG might increase the uptake of DOX by tumor tissues through suppressing the expression of P-gp. Meanwhile, compared with the control and free DOX groups, the levels of IL-10 in PEI-DOX/EGCG/FA group were significantly decreased to (40.75 ± 5.24) pg/mL and the levels of TNF-α and IFN-γ were significantly increased to (36.46 ± 1.50) and (45.39 ± 3.07) pg/mL (*p <* 0.01). At the same time, the tumor tissue showed the same trend as the serum (Figure 6(F)–(H)). It was not only demonstrated that EGCG improved the efficacy of DOX by inhibiting the expression of P-gp, but also proved that the targeting ability of PEI-DOX/EGCG/FA has significantly improved by FA modification, which promoted the anti-tumor immune response of the organism effectively.

### 3.13. *In vivo* expression of P-gp

The level of P-gp expression *in vivo* was analyzed by Western Blot. As shown in Figure 6(A)–(B), after treated with DOX, the expression level of P-gp in tumor tissues has significantly increased than control group (*p* < 0.05), which indicated that DOX can induce P-gp expression in tumor cells, further leading to drug resistance of tumor cells. Meanwhile, the expression of P-gp protein in mice treated with PEI-DOX/EGCG/FA was significantly reduced compared with control (*p* < 0.05), which indicated that EGCG in P-gp suppression together with FA in active targeting facilitated the MDR reversal and high anti-tumor efficacy of PEI-DOX/EGCG/FA.

### 3.14. *In vivo* cardiotoxicity assay

#### 3.14.1. Cardiac damage and oxidative indicators

**Figure 7.**
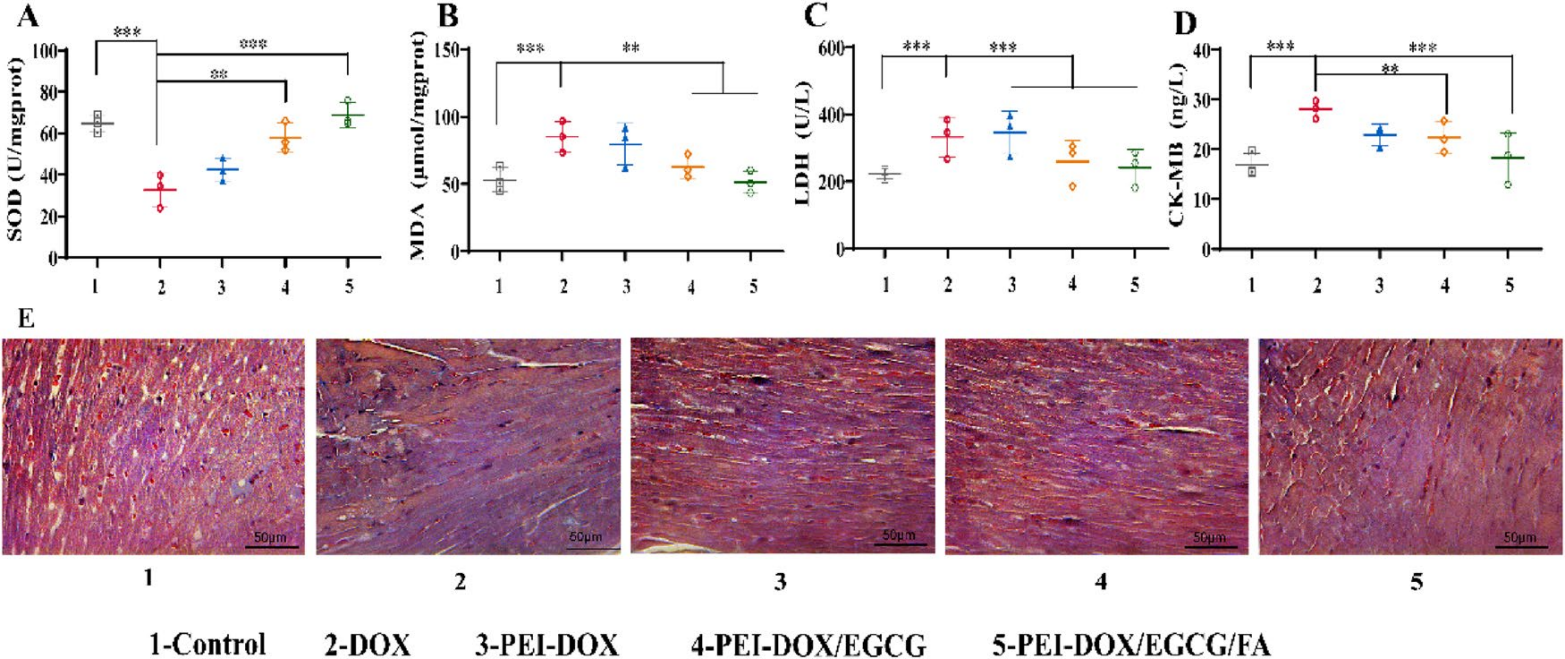
(A) Expression of SOD from heart tissue after treated. (B) Expression of MDA from heart tissue after treated. (C) Expression of LDH from serum after treated. (D) Expression of CK-MB from serum after treated. (E) Masson trichrome staining of heart tissue. All images share the same scale bar of 50 μm. ***p < 0.01, **p < 0.05.

ELISA kits were used to detect the damage and oxidative indicators in heart tissue. As shown in Figure 7(C)–(D), after treated with DOX, the level of CK-MB and LDH has increase to (27.78 ± 2.54) and (377.93 ± 65.20) ng/L, which has increased significantly compared with control ((16.28 ± 2.06) and (226.78 ± 27.53) ng/L) (*p* < 0.01). CK-MB and LDH levels in heart tissue were significantly increased compared the free DOX group (*p* < 0.01), confirming myocardial injury by free DOX. By contract, the level of CK-MB and LDH has decreased to (22.39 ± 3.50) U/L and (275.50 ± 50.90) U/L respectively in PEI-DOX/EGCG group, which show much lower damage to heart tissue than free DOX due to the ability of EGCG to remove oxygen free radicals. Therefore, after treated with PEI-DOX/EGCG/FA, the level of CK-MB and LDH has significantly decreased by EGCG and active targeted FA (*p <* 0.01).

As Figure 7(A)–(B) showed that the level of SOD decreased to (32.86 ± 7.16) U/mgprot and MDA increased to (113.24 ± 10.01) μmol/mgprot in heart tissue after treated with DOX, which has significantly changed compared with the control group (*p* < 0.01). Meanwhile, the level of SOD and MDA treated with PEI-DOX/EGCG and PEI-DOX/EGCG/FA has not changed a lot. These indicated that PEI-DOX/EGCG/FA could not only inhibit the elevated of MDA caused by DOX, but also kept SOD vitality at a high level, which could reduce the oxidative damage of heart tissue.

#### 3.14.2. Myocardial tissue fibrosis condition

As Figure 7(E) shown that after free DOX treated, myocardial tissue showed strong fibrosis (blue area) (Yildirim et al., 2023). At the same time, no obvious fibrosis was observed in PEI-DOX/EGCG and PEI-DOX/EGCG/FA group, which indicating that EGCG could remove oxygen free radicals in the body and reducing the cardiotoxicity of DOX in a further step.

## 4. Conclusion

In this study, we developed a PEI-DOX/EGCG nanoparticle based on PEI-DOX and EGCG, which not only reversed MDR and enhanced the anti-tumor effect of DOX to drug resistant cancer cells, but also reduced cardiotoxicity to myocardial cells. Furthermore, FA-PEG was also synthesized and introduced to get the active targeting PEI-DOX/EGCG/FA system. It can be seen from experiments of *in vivo* anti-tumor activity that PEI-DOX/EGCG/FA showed a strong ability of MDR reversal and active targeting, resulting an enhanced therapeutic efficacy of DOX. At the same time, the result of Western Blot revealed that EGCG could reverse drug resistant by inhibiting the expression of P-gp. It also showed the ability of EGCG to remove oxygen free radicals effectively which prevent the cardiotoxicity of DOX by cardiotoxicity assay.
